# Supplementary material for: Integron gene cassettes harboring novel variants of d-alanine-d-alanine ligase confer high-level resistance to d-cycloserine
Source: Sci Rep. 2020 Nov 26;10:20709. doi: 10.1038/s41598-020-77377-4 (PMC7691350; doi:10.1038/s41598-020-77377-4)
Supplement: Supplementary file 1 — Supplementary Information 1. [file 41598_2020_77377_MOESM1_ESM.pdf]

Supplementary Information for;

Integron Gene Cassettes Harboring Novel Variants of D-Alanine-D-Alanine Ligase Confer High-level Resistance to D-Cycloserine

Md. Ajijur Rahman[1,2], Frank Kaiser[1], Shirin Jamshidi[3], Marta Freitas Monteiro[1], Khondaker Miraz Rahman[3], Peter Mullany[1] and Adam P. Roberts[4,5]

[1]Department of Microbial Diseases, University College London, 256 Gray's Inn Road, London WC1X 8LD, UK

[2]Department of Pharmacy, University of Rajshahi, Rajshahi-6205, Bangladesh

[3]School of Cancer and Pharmaceutical Science, King's College London, London SE1 9NH, UK

[4]Department of Tropical Disease Biology, Liverpool School of Tropical Medicine, Liverpool, UK

[5]Centre for Drugs and Diagnostics, Liverpool School of Tropical Medicine, Liverpool, UK

Supplementary Table S1. Top 10 structurally homologous protein of Ddl6 and Ddl7 in PDB. The I-TASSER models of Ddl6 and Ddl7 were used to compare the structural similarity with the PDB structures.

[a] Ranking of proteins is based on TM-score of the structural alignment between the query structure (Ddl6/Ddl7) and known structures in the PDB library;
[b] RMSD is the RMSD between residues that are structurally aligned by TM-align;
[c] % identity is the percentage sequence identity in the structurally aligned region

**Rank [a]**	**PDB hits**	**Resolution (Å)**	**Protein name, size and the host**	**TM-score**		**RMSD[b]**		**% identity [c]**	
				Ddl6	Ddl7	Ddl6	Ddl7	Ddl6	Ddl7
**1**	3i12	2.2	DdlA of *Salmonella enterica* subsp. *enterica* serovar Typhimurium str. LT2 (364 aa)	0.943	0.966	1.82	1.20	28.44	28.74
**2**	4L1K	2.3	Ddl of *Xanthomonas oryzae* pv. Oryzae (384 aa)	0.910	0.922	1.59	1.30	30.54	30.54
**3**	4FU0	2.35	VanG (D-ala-D-ser ligase) of *Enterococcus faecalis* (351 aa)	0.901	0.906	1.99	1.79	31.52	31.52
**4**	1EHI	2.38	D-ala-D-lac ligase (LmDdl2) of vancomycin-resistant *Leuconostoc mesenteroides* (377 aa)	0.907	0.890	2.45	2.68	29.14	29.14
**5**	3TQT	1.88	Ddl of *Coxiella burnetii*	0.886	0.596	1.89	1.71	27.63	27.63
**6**	1E4E	2.5	VanA (D-ala-D-lac ligase) of *E. faecium* BM4147 (343 aa)	0.885	0.882	2.25	2.15	28.10	28.10
**7**	3SE7	3.07	VanA, metagenomics (346 aa)	0.872	0.876	2.02	1.81	26.43	26.43
**8**	2ZDH	1.9	Ddl of *Thermus thermophilus* HB8 (319 aa)	0.867	0.859	1.91	2.09	31.53	31.53
**9**	2I8C	2.46	Ddl of *S. aureus* (358 aa)	0.852	0.858	2.49	2.68	26.13	26.13
**10**	1IOW	1.9	Y216F mutant of DdlB of *E. coli* (306 aa)	0.812	0.812	1.90	2.02	35.35	35.69

Supplementary Table S2. Calculated energy contributions to form the Ddl6-D-cycloserine in wild and mutant complexes (kcal/mol) with standard errors of the mean (in parentheses); since the ligand was released from the receptor after around 20ns in mutant W259C form of Ddl6, so MMPBSA/MM-GBSA calculations were performed immediately before the ligand release.

**Energy distributions**	**Ddl6-D-cycloserine**	**Ddl6(W259C)-D-cycloserine**
ΔE_{ele}	-28.63(4.31)	-18.68(3.73)
ΔE_{vdw}	-15.15(2.42)	-13.04(0.83)
ΔE_{sol}	29.32(2.56)	19.57(0.10)
ΔG_{PB}	-14.47(1.81)	-12.15(1.84)
ΔG_{GB}	-12.67(1.92)	-9.06(1.37)

Supplementary Table S3. Binding free energy (ΔG) and GOLD fitness score of apigenin, quercetin and salvicine for the D-alanine and ATP binding sites of native Ddl6 and its two single mutants.

	**D-alanine binding site**					
	**Quercetin**		**Apigenin**		**Salvicine**	
	ΔG (kcal/mol)	Score	ΔG (kcal/mol)	Score	ΔG (kcal/mol)	Score
**Ddl6**	-40.20	33.42	-43.41	35.48	-43.81	41.76
**L164F**	-39.90	32.33	-44.61	37.21	-44.87	41.95
**W259C**	-39.85	32.95	-41.58	33.02	-44.93	41.58
**DdlTd**	-40.20	33.43	-39.90	32.34	-40.14	39.13
	**ATP binding site**					
**Ddl6**	-34.12	25.30	-27.29	25.09	-39.70	35.20
**L164F**	-35.96	33.98	-29.76	29.69	-46.95	38.99
**W259C**	-37.84	34.52	-30.55	29.78	-39.54	37.28
**DdlTd**	-31.57	24.89	-31.98	27.04	-38.27	36.66

Supplementary Table S4. Primers used in this study

Name	**Primer Sequence (5'-3')**	Target	Source
TDIF	TCAAGCCAAAATCAGGCTCT	*intI* in forward direction	[1]
MARS2	GCAATGTCAGGTTGAAGC	*attC* in reverse direction	[1]
ddlF	GTAGTACTTGCTGGAGGAT TAA	*ddl6/ddl7* (Forward primer)	This work
ddlR	GCCTTCAATTTTTATTGCAT TATGT	*ddl6/ddl7* (Reverse primer)	This work
TddlF	GCTA*GGATCC*ATGAAGGTA GTAGTACTTGC	*ddl6/ddl7* (Forward primer, *Bam*HI site underlined)	This work
TddlR	ACGC*TCTAGA*TTAGCCTTCA ATTTTTATTGC	*ddl6/ddl7* (Reverse primer, XbaI site underlined)	This work
Tddl28aR	GCGG*CTCGAG*TTAGCCTTC AATTTTTATTGC	*ddl6/ddl7* (Reverse primer, XhoI site underlined)	This work
Ddl6-490F	TATCAGAAGTTAATTTTGAT ACAATAAAGAGAGA	*ddl6* cloned into pGEM-T Easy (forward primer to change C of *ddl6* at c.490 to T)	This work
Ddl6-490R	ATGAATAAGTTTTCCATTTT GCTGTAG	*ddl6* cloned into pGEM-T Easy (reverse primer to couple with *Ddl*6-490F primer)	This work
Ddl6-777F	CAACGAAATATGTCCTGCTG AAATA	*ddl6* cloned into pGEM-T Easy (forward primer to change G of *ddl6* at c.777 to T)	This work
Ddl6-777R	GATAATCCTTTTTGATACTT ATTTTTATAATCATAAAAT	*ddl6* cloned into pGEM-T Easy (reverse primer to couple with *Ddl*6-777F primer)	This work
Upint-5100F	GGCGGAGATGAAGATACCC TT	2.7 kb upstream of integrase of *T. denticola ATCC35404*	This work
Upint-3685F	TCACAGCCTGCAAGCAATG T	4.2 kb upstream of integrase of *T. denticola ATCC35404*	This work
Upint-721F	GGTGGGGAGCTCATCATAA A	7.2 kb upstream of integrase of *T. denticola ATCC35404*	This work
Upint-122F	GCTTCCAAATCTATACCGGA AC	7.8 kb upstream of integrase of *T. denticola ATCC35404*	This work
EC-ddlA-F	GCTA*GGATCC*ATGGAAAAA CTGCGGGTAGGAAT	*ddlA* of *E. coli* (forward primer, *Bam*HI site underlined)	This work
EC-ddlA-R	ACGC*AAGCTT*CATTGTGGTT TTCAATGCGTTATC	*ddlA* of *E. coli* (reverse primer, HindIII site underlined)	This work
DdlTD-F	GCGC*GAATTC*ATGAATATAG CAATCATTTACGGCGG	*ddl* of *T. denticola* (forward primer, *Eco*RI site underlined)	This work

DdlTD-R	ACGC*AAGCTT*AGATTGACG GCAGGTTTTGAGTTTTC	*ddl* of *T. denticola* (reverse primer, HindIII site Underlined)	This work
M13F	GTAAAACGACGGCCAG	M13 forward sequencing	Universal
M13R	CAGGAAACAGCTATGAC	M13 reverse sequencing	Universal

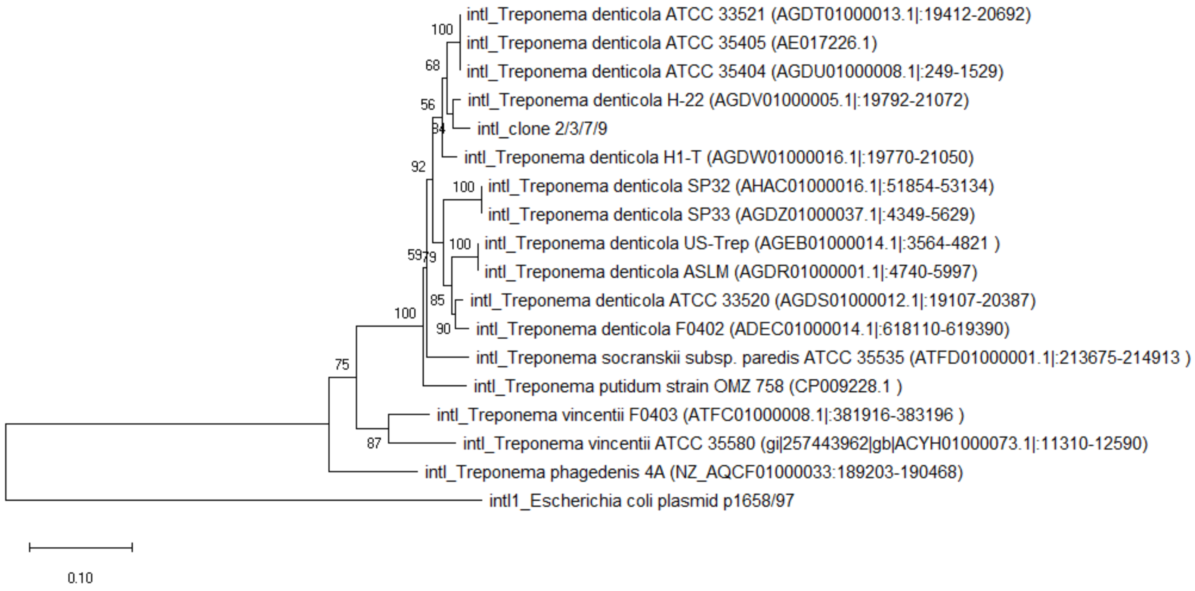

Supplementary Fig. S1. The phylogenetic tree of homologues of *intI* found upstream of *ddl7* in the 4421-bp inserts in pGEM-T Easy. The evolutionary relationship was inferred using Neighbour-Joining Method [2]. The percentage of replicate trees in which the associated taxa clustered together in the bootstrap test (1000 replicates) are shown next to the branches. Evolutionary analyses were conducted in MEGA X[3]. *intI1* of *E. coli* was used as outgroup.

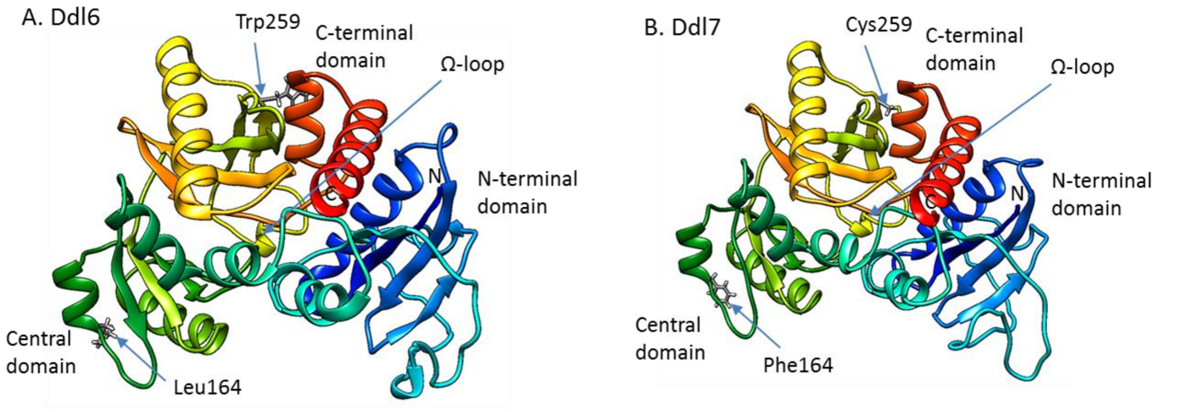

Supplementary Fig. S2. Predicted 3D-structures of Ddl6 (A) and Ddl7 (B) monomer as determined by I-TASSER. The model was viewed by UCSF Chimera. The residues at 164 and 259 are shown. The N-terminal domain and C-terminal domains were shown in blue and red colour, respectively.

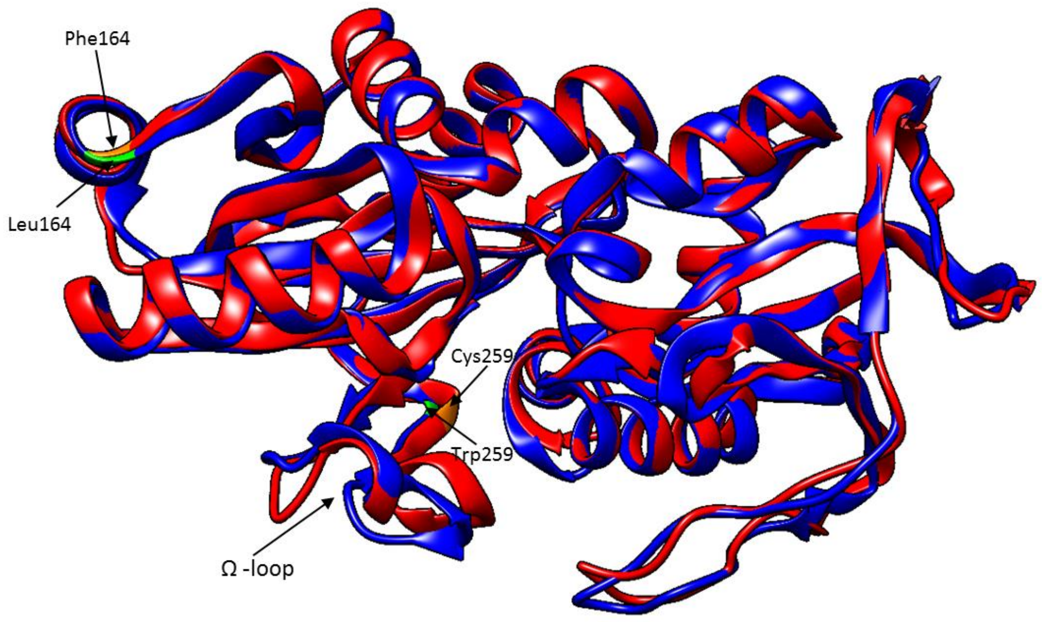

Supplementary Fig. S3. Superimposition of predicted 3D models of Ddl6 (blue) and Ddl7 (red). The alignment of the 3D structures of Ddl6 and Ddl7 were done using TM-align program (http://zhanglab.ccmb.med.umich.edu/TM-align/).

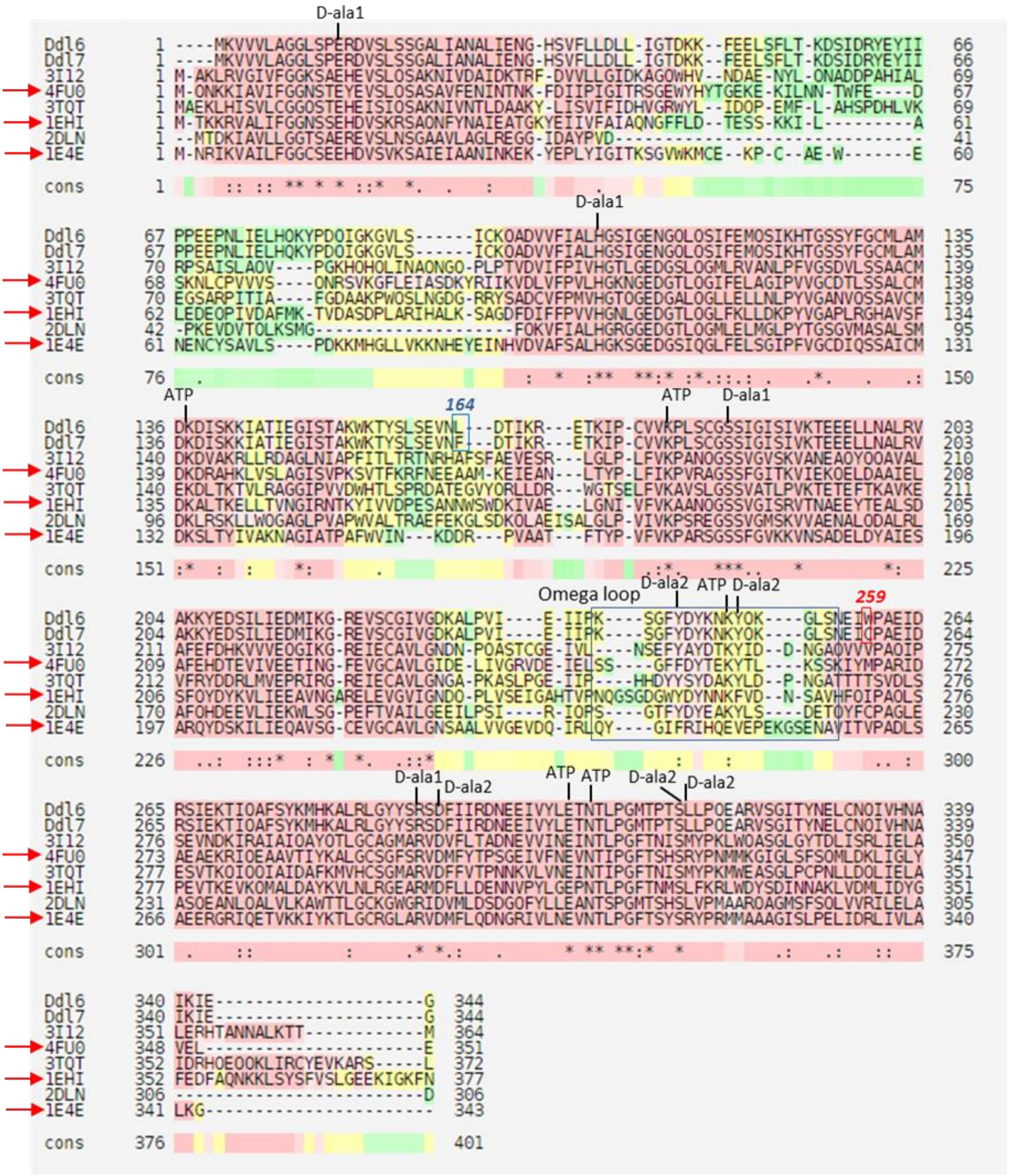

Supplementary Fig. S4. Multiple sequence alignment of the four proteins in PDB which have highest structural similarity to Ddl6 and Ddl7 (PDB codes: 3I12, 4FU0, 3TQT and 1EHI). The *E. coli* DdlB (PDB code: 2DLN) and *E. faecium* VanA (PDB code: 1E4E) were included in the alignment to compare the active/binding sites. PDB code 3I12: Ddl of *S. enterica* subsp. enterica Serover Typhimurium Str. LT2; 4FU0: D-ala-D-ser ligase (VanG) of E. faecalis; 3TQT: Ddl of *Coxiella burnetii*; 1EHI: D-ala-D-lac ligase of *L. mesenteroides* (LmDdl2). The vancomycin resistant proteins are marked with red arrows.

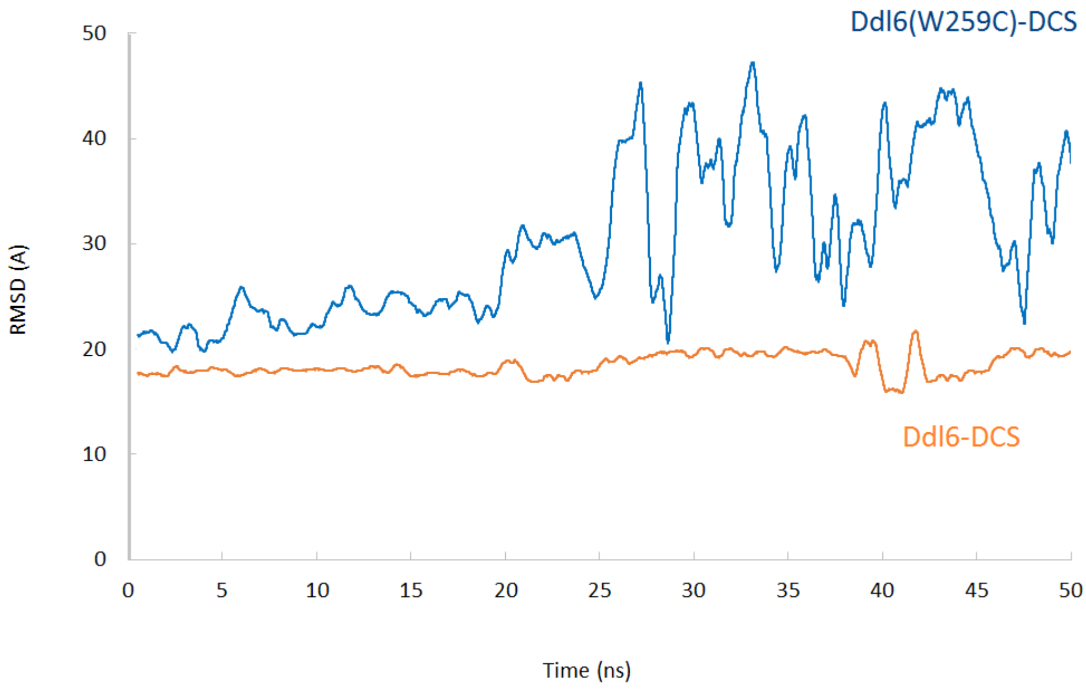

Supplementary Fig. S5. Monitoring the average movement of distance between the center of D-cycloserine and residue 259 (W259 and C259 of wild and mutant types respectively) in each complex during MD simulations.

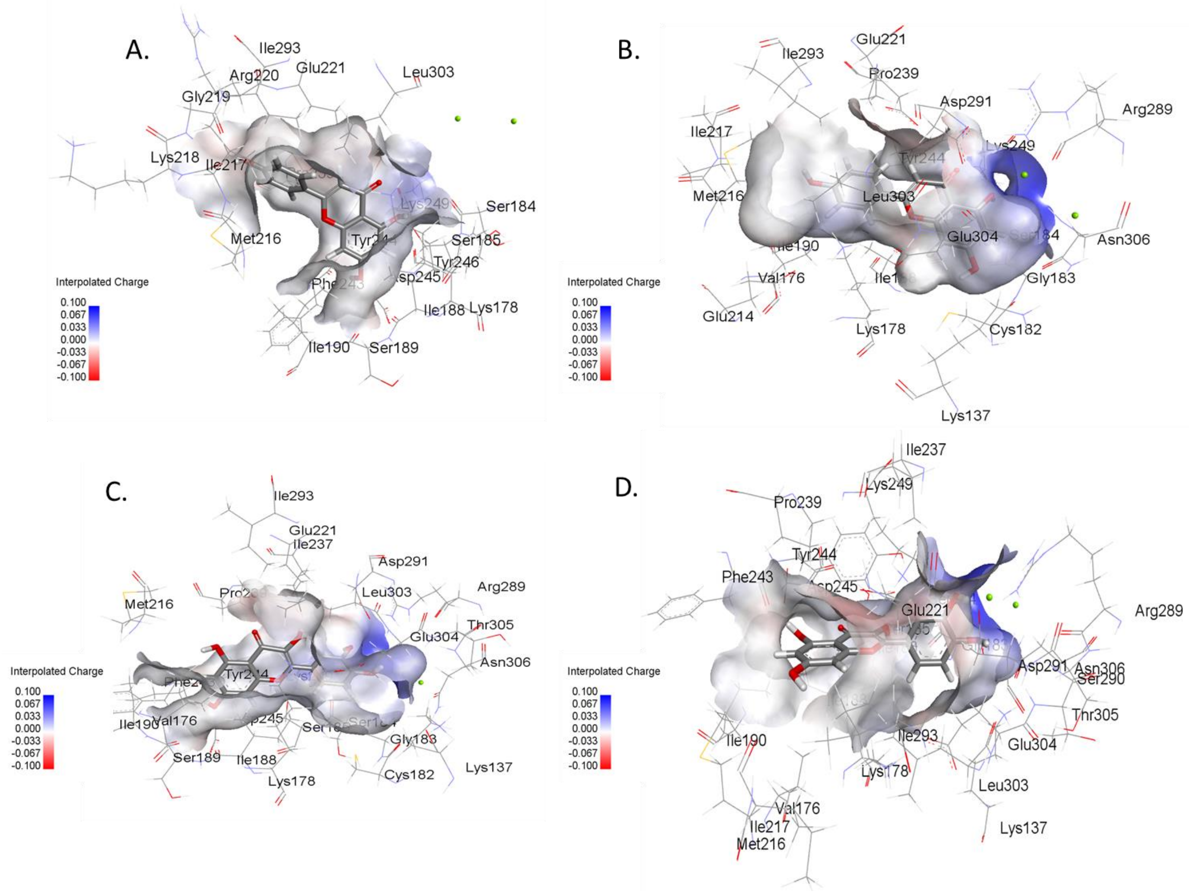

Supplementary Fig. S6. 3D structures of interaction of apigenin and quercetin with the ATP binding sites of WT Ddl6 and W259C mutant. A) Apigenin with WT Ddl6, B) Apigenin with W259C mutant, D) Quercetin with WT Ddl6 and D) Quercetin with W259C mutant.

References:

1 Tansirichaiya, S., Rahman, M. A., Antepowicz, A., Mullany, P. & Roberts, A. P. Detection of Novel Integrons in the Metagenome of Human Saliva. *PloS one* **11**, e0157605, doi:10.1371/journal.pone.0157605 (2016).

2 Saitou, N. & Nei, M. The neighbor-joining method: a new method for reconstructing phylogenetic trees. *Mol Biol Evol* **4**, 406-425 (1987).

3 Kumar, S., Stecher, G., Li, M., Knyaz, C. & Tamura, K. MEGA X: Molecular Evolutionary Genetics Analysis across Computing Platforms. *Molecular Biology and Evolution* **35**, 1547-1549, doi:10.1093/molbev/msy096 (2018).
