# Supplementary material for: Integron gene cassettes harboring novel variants of d-alanine-d-alanine ligase confer high-level resistance to d-cycloserine
Source: Sci Rep. 2020 Nov 26;10:20709. doi: 10.1038/s41598-020-77377-4 (PMC7691350; doi:10.1038/s41598-020-77377-4)
Supplement: Supplementary file 2 — Supplementary Video Legends. [file 41598_2020_77377_MOESM2_ESM.docx]

**Legend for Supplementary Video 1 and 2**

Supplementary Video 1 shows that D-cycloserine, in complex with the wild-type protein Ddl6, remains stable in the binding site during the course of the MD simulations whereas in supplementary video 2 we see D-cycloserine in complex with the W259C mutant protein is released from the recombinant protein. Monitoring the ligand that is released from the binding site could be observed using the Surface format in the mutant version of the protein (supplementary video 2). However since the ligand stays in the binding site in the course of MD simulations for the wild type Ddl6, and can't be seen from any of the angles using Surface format to make the supplementary video 1, the video was generated with a Ribbon type format for the sake of observing the ligand in the complex structure of the wild type Ddl6.
